# Postoperative Mechanomodulation Decreases T-Junction Dehiscence After Reduction Mammaplasty: Early Scar Analysis From a Randomized Controlled Trial

**DOI:** 10.1093/asj/sjad269

**Published:** 2023-08-22

**Authors:** Jasmine Panton, Nicole Vingan, Jennifer Barillas, Yucel Akgul, Ariane Lazzarini, Christopher J Coroneos, Bardia Amirlak, Jeffrey Kenkel, Abby Culver

## Abstract

**Background:**

Soft tissue and cutaneous tension is an important contributor to complicated wound healing and poor scar cosmesis after surgery and its mitigation is a key consideration in aesthetic and reconstructive procedures.

**Objectives:**

The study objective was to assess the efficacy of the force modulating tissue bridge (FMTB) (“Brijjit”, Brijjit Medical Inc., Atlanta, GA) in reducing mechanical tension on postoperative wounds.

**Methods:**

A prospective, single-center, randomized, within-subject clinical trial was conducted to evaluate wound healing and nascent scar formation after 8 weeks of postoperative wound support with the FMTB. Patients received standard of care (SOC) subcuticular closure on the vertical incision of 1 breast and experimental closure with the FMTB on the contralateral incision after Wise-pattern reduction mammaplasty. Three-dimensional wound analysis and rates of T-junction dehiscence were evaluated by clinical assessment at 2, 4, 6, and 8 weeks postsurgery.

**Results:**

Thirty-four patients (n = 68 breasts) completed 8 weeks of postoperative FMTB application. There was a reduced rate of T-junction wound dehiscence in FMTB breasts (n = 1) vs SOC breasts (n = 11) (*P* < .01). The mean vertical incision wound area during the intervention period was significantly decreased in the FMTB breast (1.5 cm^2^) vs the SOC breast (2.1 cm^2^) (*P* < .01) and was significantly lower at 2-, 4-, and 8-week follow-up (*P* < .01). Only the closure method was significantly associated with variations in Week 8 wound area (*P* < .01) after linear regression modeling.

**Conclusions:**

FMTBs decrease nascent scar dimensions and reduce the occurrence of wound dehiscence. This study provides evidence that the use of continuous mechanomodulation significantly reduces postoperative wound complications after skin closure.

**Level of Evidence: 2:**

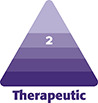

See the Commentary on this article here.

Mechanical and tensile forces play an important role in postoperative wound healing.^1,2^ Plastic surgeons are trained to mitigate tensile forces in aesthetic and reconstructive procedures, incising skin along Langer's lines^3,4^ or optimizing fascial and soft tissue closure.^5^ In the last few decades, hallmark preclinical studies have outlined the clinical effects of mechanomodulation, demonstrating that the translation of mechanical forces into chemokine-regulated inflammatory pathways induces fibrosis.^2^ These forces can become aberrantly upregulated, leading to pathologic scar formation such as keloid or hypertrophic scarring. Modulating these mechanical forces significantly reduces scar formation and decreases the propensity for pathologic scars in vitro and in vivo.^1,6^ Cutaneous adjuncts such as silicone gel sheets or tape have been used to improve postoperative scar cosmesis.^6-9^

The relationship between mechanical tension and wound healing is circular. Mechanical stress both encourages dermal tissue remodeling and is a key contributor to the gradual increase in the tensile strength of the nascent scar after injury.^10,11^ In the first weeks of healing after surgery, a new matrix of collagen and extracellular matrix proteins reorganizes in response to injury and yields 50% and 80% of the tensile strength of unwounded skin by 4 and 6 weeks after surgery, respectively.^12^ This gain in the scar's tensile strength is driven by a response to tension, and studies have shown the absence of mechanical stress impairs normal wound healing pathways. Conversely, aberrant or excessive mechanical tension on healing wounds can lead to dehiscence or chronicity in the short term and pathologic scarring in the long term. Thus, strategies or adjuncts to attenuate cutaneous tension play an important role in decreasing wound complications as well.^13^

When tissues are reapproximated after surgical incisions, tensile strength is concentrated at the interface between suture and tissue.^14^ As absorbable sutures typically begin to lose strength around 2 weeks after surgery, this tension is transferred to the wound itself, correlating with the time point at which wound dehiscence is first observed.^11,15^ Thus, decreasing tissue strain postoperatively with cutaneous application of force modulating tissue bridges (FMTBs) potentially mitigates this translation of tensile forces and better supports postoperative wound healing.

The FMTB (“Brijjit”, Brijjit Inc., Atlanta, GA) is a novel, FDA-approved device for wound closure and support. The device is applied externally and perpendicular to a wound or incision, decreasing cutaneous tension through a process known as “mechanomodulation.”^16^ Its biomechanical efficacy was first described in 2018 by Kazmer and Eaves.^17^ The authors found that FMTBs significantly decreased transverse tissue strain in computer-generated modeling compared with the standard-of-care (SOC) closure technique which utilizes sutures. According to their analysis, the compressive stresses at the tissue-suture interface were 4000 mmHg, while those at the tissue-FMTB interface were 20 mmHg.^17^

In this study, we report clinical findings on postoperative wound healing and early scar formation from a randomized controlled trial in which the FMTB is used to offload mechanical tension on postoperative incisions. Taking Wise-pattern reduction mammaplasties as a clinical and surgical model, we used objective endpoints to demonstrate the efficacy in mitigating wound healing complications and supporting nascent scar formation.

## METHODS

Our study is a single-center, prospective, randomized controlled trial conducted at a large academic center. The study was approved by the UT Southwestern Medical Center Institutional Review Board. Participants to date include 34 females undergoing bilateral reduction mammaplasty with a modified Wise-pattern resection for treatment of symptomatic macromastia. Procedures were performed by 3 surgeons at a single outpatient surgery center. Inclusion criteria (Table 1) included healthy adult females between 18 and 70 years of age planning to have a bilateral breast reduction with Wise-pattern skin resection to treat symptomatic macromastia. Patients also had to be able to adhere to FMTB therapy and attend biweekly follow-up visits through the 8-week postoperative period and long-term follow-up visits at 3 6, and 12 months following surgery. Exclusion criteria included known allergies or sensitivities to general adhesives or adhesive tape, the use of isotretinoin or systemic steroids within the previous year, individuals with significant scarring or clinically significant preoperative breast asymmetry, individuals who were malnourished or had a BMI ≥40 kg/m^2^, individuals with a known history of breast cancer or previous radiation therapy, active smokers, or individuals known to have a disorder that negatively affected wound healing.

**Table 1.** Study Inclusion and Exclusion Criteria

Inclusion criteria	Exclusion criteria
Healthy adult females 18-70 years of age Planned procedure is bilateral breast reduction with modified Wise (anchor, inverted “T”) scar pattern Lateral incision measuring >5 cm (for additional FMTB application on lateral incision) Ability to adhere to wound therapy after surgery for 8 weeks or have a willing family member/partner to assist with wound therapy care Willing to follow wound care therapy as instructed by study staff Willing to return for follow-up visits and undergo study evaluations	Diagnosed with known allergy to general adhesives/adhesive tape History of using the following prescription medications: − Accutane within the past year − Systemic steroid use within the past year Significant scarring on the test site/area(s) Malnutrition BMI >40 kg/m^2^ History of radiation therapy History of breast cancer Active smokers Any disorder known to negatively affect wound healing (eg, autoimmune disease, connective tissue disease) Observable preoperative or intraoperative breast asymmetry that, in the investigator's opinion, would interfere with the evaluation of the efficacy of the wound therapy

FMTB, force modulating tissue bridge.

**Figure 1.**
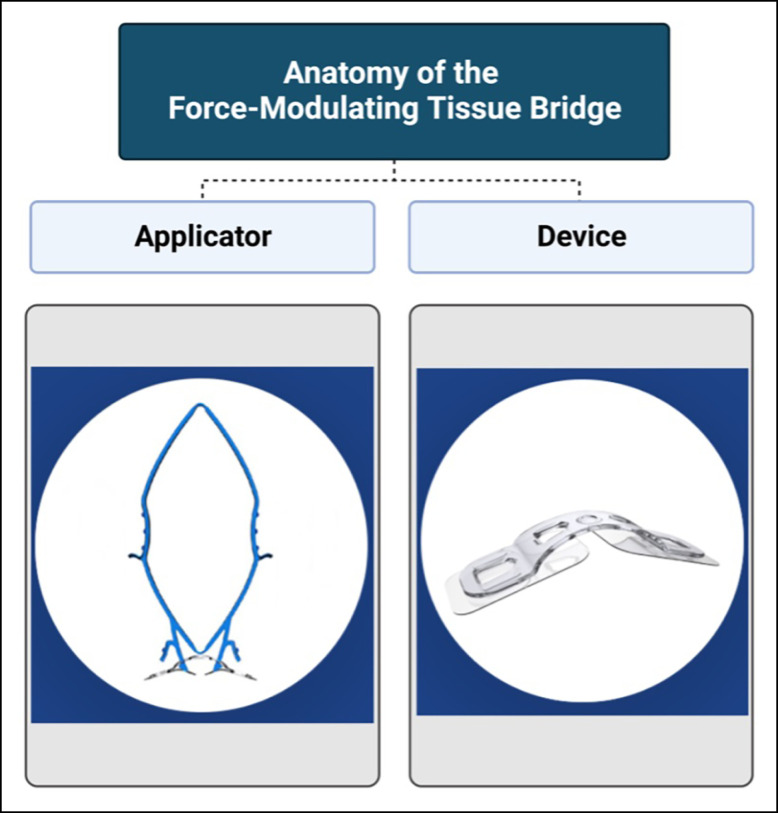
Force modulating tissue bridge applicator and device.

**Figure 2.**
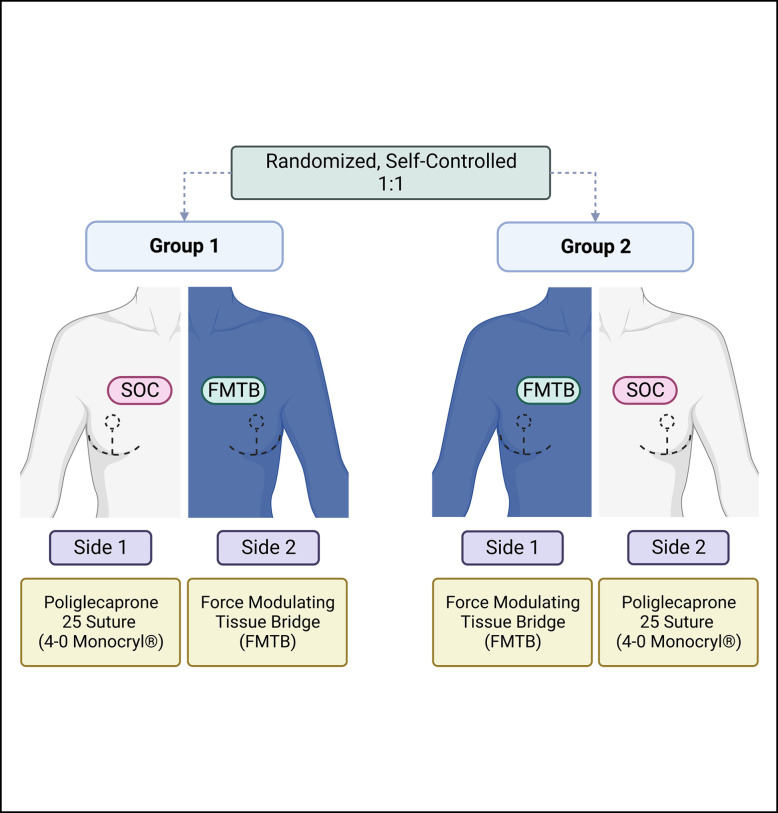
Subject randomization and group allocation workflow. Patients were self-controlled to account for innate differences in wound healing and scar quality. FMTB, force modulating tissue bridge; SOC, standard of care. Figure created using Biorender.com.

Subjects in this postoperative intervention period analysis were enrolled between October 2021 and April 2023. Patients were self-controlled by means of a bilateral control vs intervention model: 1 breast underwent final skin closure with absorbable subcuticular sutures and the contralateral breast underwent final skin closure with FMTBs (Figure 1). Patients eligible for the study were prospectively randomly allocated into 2 groups prior to study commencement by a web-based, open-source randomizer.^18^ Randomization assignments were concealed until just prior to surgery when study investigators provided the assignment to the circulating and scrub nurses. The surgeon was blinded to the subject's assignment until final skin closure at the end of the procedure. Both breasts underwent dermal closure by the buried, inverted interrupted technique with 3-0 Monocryl suture (Poliglecaprone 25/3-0 Monocryl; Ethicon, Inc., Raritan, NJ). This was followed by application of the FMTB in the experimental breast or the running subcuticular technique with 4-0 Monocryl in the control breast, for final superficial tissue approximation (Figure 2).

After intraoperative procedural symmetry was confirmed, final vertical incision closure took place with sutures or FMTBs, depending on assignment. (Video).

**Figure 3.**
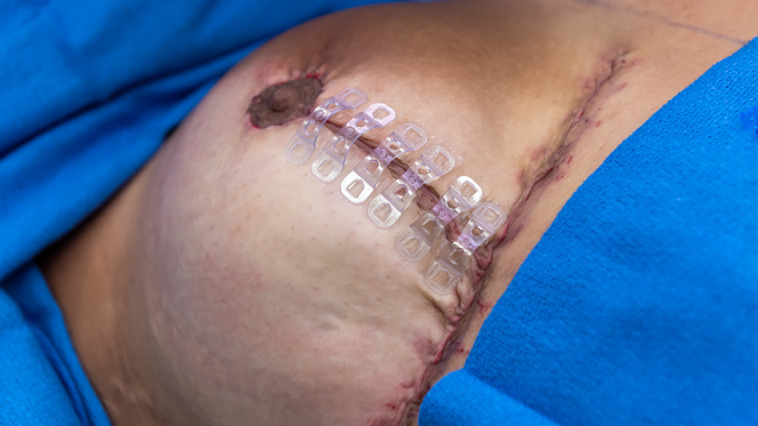
Force modulating tissue bridge placement along vertical incision of 27-year-old female patient after Wise-pattern reduction mammaplasty. Following inverted interrupted dermal closure with 3-0 Monocryl (Ethicon, Raritan, NJ) along the vertical incision, force modulating tissue bridges were applied for final tissue approximation and superficial closure on the experimental breast.

**Figure 4.**
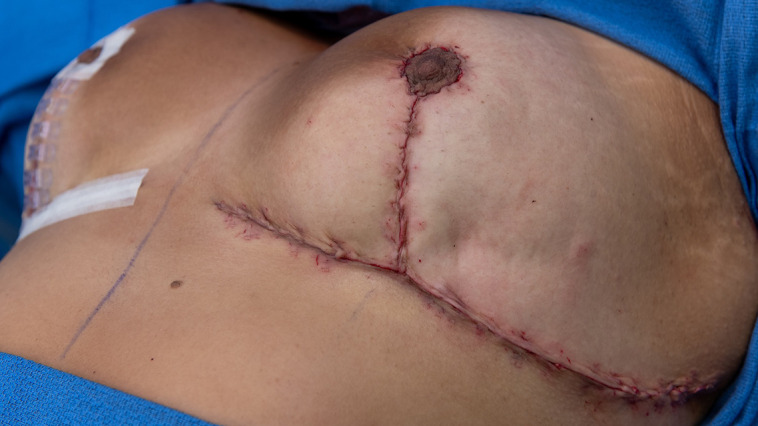
A 27-year-old female patient undergoing Wise-pattern reduction mammaplasty with stand-of-care closure shown. In the control breast, inverted interrupted dermal closure was completed with 3-0 Monocryl (Ethicon, Raritan, NJ) suture along the vertical incision. Afterward, 4-0 running subcuticular Monocryl sutures were used for final tissue approximation and superficial closure on the contralateral incision, serving as a within-subject control.

Group 1 underwent final skin closure using 4-0 Monocryl for Side 1 (patient right breast) and FMTB for Side 2 (patient left breast). Group 2 underwent final skin closure with FMTB for Side 1 and Poliglecaprone 25 suture for Side 2 (Figures 3, 4). Aside from final skin approximation and closure, the remainder of the tissue closure technique was identical for both breasts, for all incisions (ie, periareolar and inframmary fold).

Patients returned at 2, 4, 6, and 8 weeks after surgery for postoperative wound therapy visits, where incisions were examined, assessments captured, and FMTBs removed and replaced by a study investigator. Subjects received instructions before surgery regarding proper application of FMTBs in case of early accidental device removal between postoperative study visits. On the morning of surgery, patients received their own FMTBs and alcohol prep pads. Instructions for postoperative hygiene were the same as for other patients undergoing reduction mammaplasty at our institution. Showering was permitted starting 48 hours postsurgery, facing away from the shower head, and patting breasts dry with a towel. Patients initiated scar care as recommended by the operating surgeon at 6 weeks postsurgery but were instructed only to treat the medial and lateral inframammary fold incisions to reduce the potential for confounding. Preliminary results are reported according to CONSORT 2010 extension guidelines for within-person randomized trials.^19^

### Study Endpoints

The primary endpoint of the complete study is clinical, objective, and subjective assessments of the vertical incision scar through 12 months after surgery based on the Patient and Observer Scar Assessment Scales (POSASs) and blinded assessment of clinical photographs by trained surgeons. Secondary endpoints of the study include histologic analysis, ultrasonographic measurements of the healing surgical site, measurements of nascent scar ultrastructure and stratum corneum integrity, colorimetry, and nascent scar area and volume, the last of which is the focus of this preliminary report. Per published guidelines, the significance of nascent scar measures was considered to be a level of α(*k* + 1).

In this study, we define the “vertical incision” as the surgical site and clinical model during the intraoperative and postoperative periods. During the 8-week postoperative wound support and intervention period, we defined the healing incision as a “nascent” or “early” scar for simplicity.

### Subject Demographics, Operative Measures, and Safety

Demographic data collected included BMI (kg/m^2^), age at surgery, race/ethnicity, and smoking status. Additional anthropometric variables were collected through chart review, including: sternal notch-to-nipple distance (cm), degree of ptosis (Grade I-IV), base width (cm), and nipple-to-inframammary fold distance (cm). Lastly, operative measures including anesthesia time, American Society of Anesthesiologists class, and resection weight (g) were recorded. These patient-level and operative variables were measured due to the association of certain variables with postoperative wound complications and morbidity, such as dehiscence and delayed wound healing. Subjects were additionally interviewed at each postoperative follow-up visit and charts were retrospectively reviewed to assess the occurrence of adverse events.

### Postoperative Nascent Scar Measurement

**Figure 5.**
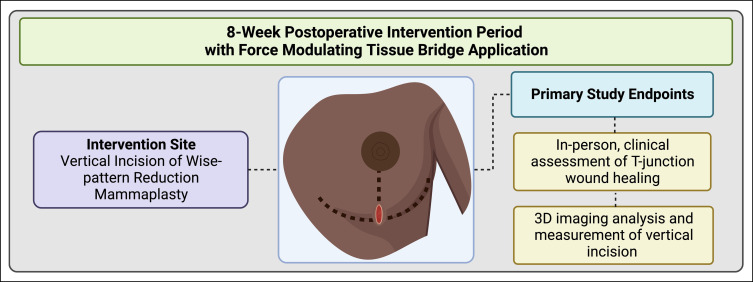
Intervention period and study endpoints. 3D, three-dimensional. Figure created with Biorender.com (Toronto, Ontario, Canada).

Healing of the vertical incision was measured by 3-dimensional (3D) imaging. Advanced wound imaging and analytics were used to measure the vertical incision 2, 4, 6, and 8 weeks postoperatively to track the progression of healing over time (Figure 5). Measurements were taken with an eKare Insight device (eKare Inc., Fairfax, VA) and included healing scar depth (cm), surface area (cm^2^), and volume (cm^3^). Ghost overlays and uniform patient positioning were employed to ensure healing incisions were captured from the same position and perspective across follow-up visits. Although the eKare system employs automatic, algorithmic tracings of active wounds, investigators also reapproximated the tracing of incisions if necessary to ensure the accuracy of tracings. The device offers moderate to high levels of intra- and interrater reliability for area and volume and is considered a validated tool for this purpose.^20,21^

### Clinical Assessment of Nascent Scar Formation and T-junction Healing

Postoperative healing of the vertical incision and T-junction were each assessed clinically. The vertical incision was defined as the treated area undergoing intervention, from the inferior pole of the nipple-areolar complex to the inframammary fold. We defined the T-junction as the immediate trifurcation of the vertical and inframammary fold incisions, measured in cm. Delayed healing at the T-junction was defined at a threshold of clinical significance, ≥1 cm × 1 cm (1 cm^2^). “Early dehiscence” was used to describe wound healing during the 2- to 8-week postoperative period when susceptibility to dehiscence is typically observed.

### Statistical Analysis

The secondary endpoint discussed in this preliminary report did not have a predetermined, powered sample size. The sample size calculation for this study was determined by the primary outcome: Observer Scar Assessment Scale Score (POSAS-O). The overall study will enroll 42 patients which will allow us to detect a moderate effect (Cohen's *d* = 0.5) in scar quality measured by POSAS-O with 80% power, accounting for an estimated 20% loss of patients. The initial power calculation included loss to follow-up, and this analysis was planned once enough patients had been recruited without the lost-to-follow-up inflation. This was decided post hoc given the challenges of performing research during the COVID-19 pandemic.

Statistics were performed by study investigators with GraphPad Prism9 (Graph Pad Software, Boston, MA) and by biostatisticians with R (R Foundation for Statistical Computing, Vienna, Austria) at the UT Southwestern Biomedical Informatics Core Facility. Shapiro-Wilk normality testing confirmed the appropriateness of parametric testing to assess 3D wound metrics; multiple paired *t*-tests were used to compare nascent scar area, volume, and maximum depth between breasts during the intervention period. The 2-stage step-up method of Benjamini, Krieger, and Yekutieli was employed to identify the potential for false discovery in our 3D measurement analysis, with a desired false discovery rate *Q* set to 1%. Multivariable linear regression was performed in post hoc analyses to assess the relationship between patient variables and variations. Univariate analysis was completed with analysis of variance and subsequent bootstrapping completed to assess for variables potentially contributing to postoperative wound area. Binomial testing was employed to assess the significance between observed and expected rates of T-junction dehiscence between treatment groups. A *P*-value <.05 was considered statistically significant.

## RESULTS

**Figure 6.**
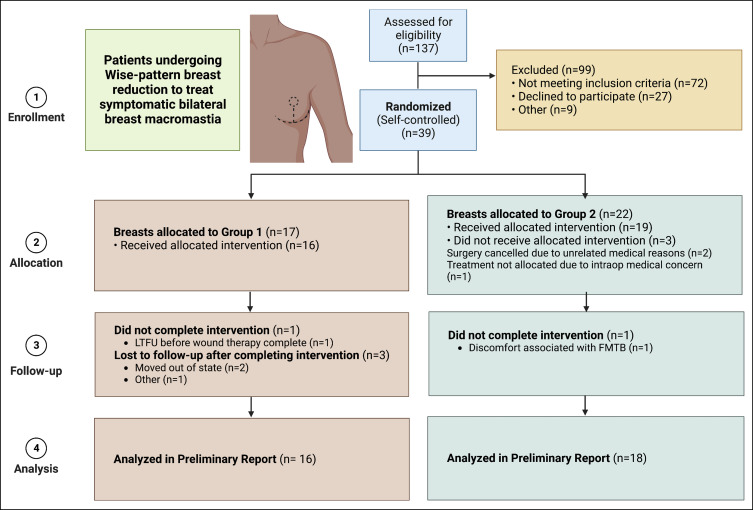
Flow diagram of clinical trial. 34 patients were included in our preliminary analysis. FMTB, force modulating tissue bridge. Figure created with Biorender.com.

**Figure 7.**
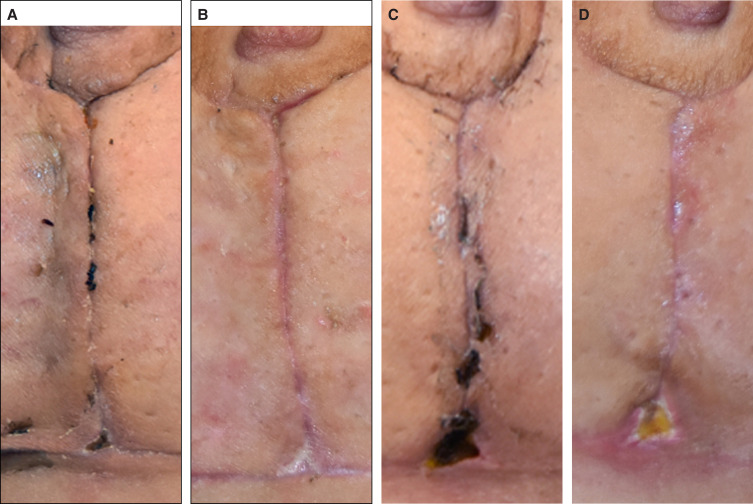
Clinical assessment of the vertical incision of a 35-year-old female patient during the postoperative intervention period. (A) Force modulating tissue bridge breast at 2-week follow-up and (B) at 4-week follow-up. (C) Standard-of-care breast at 2-week follow-up and (D) at 4-week follow-up.

**Figure 8.**
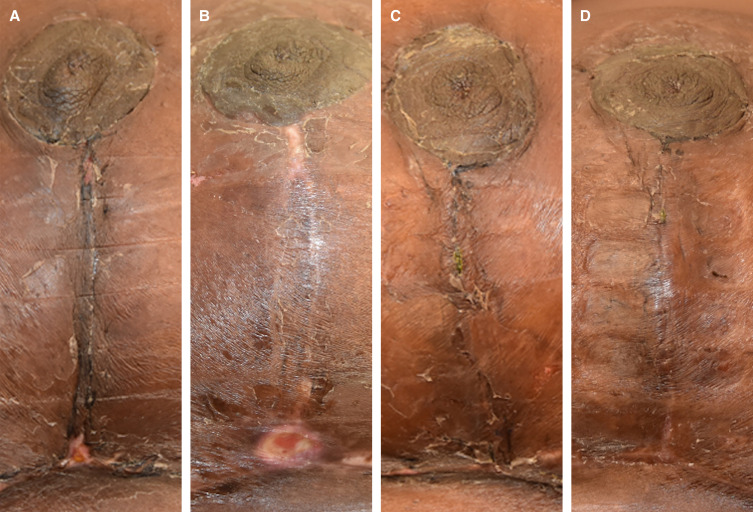
Clinical assessment of the vertical incision of a 34-year-old female patient during the postoperative intervention period. (A) Standard-of-care breast at 2-week follow-up and (B) at 4-week follow-up. (C) Force modulating tissue bridge breast at 2-week follow-up and (D) at 4-week follow-up.

**Figure 9.**
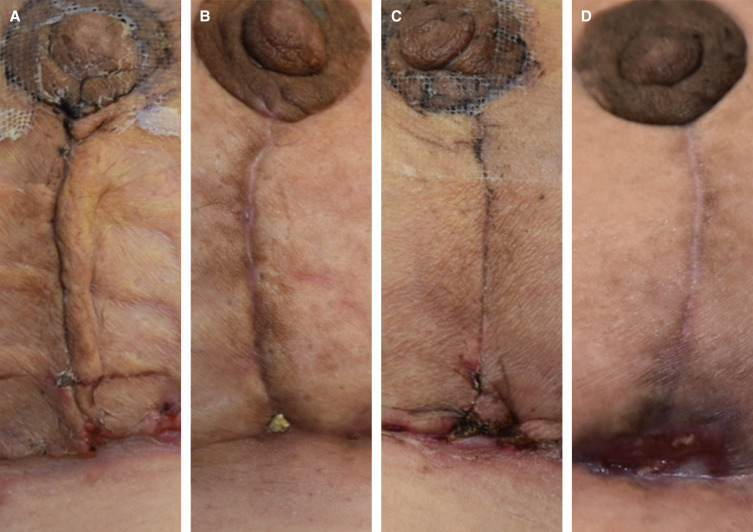
Clinical assessment of the vertical incision of a 36-year-old female patient during the postoperative intervention period. (A) Force modulating tissue bridge breast at 2-week follow-up and (B) at 8-week follow-up. (C) Standard-of-care breast at 2-week follow-up and (D) at 8-week follow-up.

Between October 2021 and March 2023, 34 patients (n = 68 breasts) were enrolled in the study and are included in this preliminary analysis of postoperative healing (Figure 6). The randomization process assigned 47% (n = 16) of patients to Group 1% and 53% (n = 18) to Group 2. In total, 34 breasts each underwent final skin closure with FMTB vs SOC closure. All of the study participants (100%, n = 34) were female. The mean [standard deviation] age of the study cohort was 42.5 [14.5] years (range, 19-70 years), and the mean BMI was 31.39 [4.2] kg/m^2^. Demographic variables, operative variables (Table 2), and anthropometric measures (Table 3) were statistically indistinct between assignment groups and breasts. All 34 patients completed 8 weeks of continuous treatment with FMTBs after surgery and underwent biweekly clinical assessment (Figures 7-9). Overall, 84% of postoperative visits occurred within the target follow-up interval (Table 4). The mean patient follow-ups for the 2-, 4-, 6-, and 8-week postoperative visits were 13.6 [2.6] days, 28.5 [9] days, 42.7 [2.9] days, and 58.0 [4.7] days, respectively. All analyses followed intent to treat.

**Table 2.** Demographic Variables Between Treatment Allocation Groups

Demographic variable	Group 1 (n = 16)		Group 2 (n = 18)		
	Mean [SD]	Range	Mean [SD]	Range	*P*-value
Age (years)	42.25 [14.21]	19-66	43.06 [14.57]	19-70	.637
BMI (kg/m^2^)	32.51 [4.69]	26.78-39.84	30.53 [3.323]	24.43-38.27	.323
	N (%)		N (%)		*P*-value
Gender					
Female	16 (47)		18 (53)		>.999
Race					.168
Asian	0 (0)		0 (0)		
Black	10 (62.5)		6 (33.3)		
White	6 (37.5)		12 (66.7)		
Ethnicity					>.999
Hispanic	1 (6.25)		2 (11.11)		
Non-Hispanic	15 (93.75)		16 (88.9)		
Smoking status					.323
Never	13 (81.25)		1 (5.56)		
Former	3 (18.75)		17 (94.4)		
Current	0 (0)		0 (0)		
Operative variables					
Pedicle					
Superomedial	16 (100)		17 (94.4)		>.999
Inferior	0 (0)		1 (5.56)		
Resection pattern					
Wise pattern	16 (100)		18 (100)		1.0
	Mean [SEM]	Range	Mean [SEM]	Range	*P-*value
Anesthesia time (minutes)	178.5 [15.93]	136-405	175.8 [12.86]	134-369	.850
ASA class	1.69 [0.15]	1-3	1.94 [0.10]	1-3	.359

ASA, American Society of Anesthesiologists; SD, standard deviation; SEM, standard error of the mean.

**Table 3:** Anthropometric Measures and Resection Weight of Treatment and Control Breasts

Anthropometric measures	FMTB (n = 34 breasts)			SOC (n = 34 breasts)			
	Mean [SEM]	Range	95% CI	Mean [SEM]	Range	95% CI	*P*-value
Sternal notch-to-nipple distance (cm)	32.85 [0.69]	25-42	31.45, 34.25	32.93 [0.71]	26-42	31.49, 34.37	.832
Nipple to inframammary fold distance (cm)	17.28 [0.60]	11-27	16.05, 18.51	17.04 [3.04]	12-23	15.98, 18.10	.409
Ptosis	2.21 [0.08]	1-3	2.04, 2.38	2.18 [0.08]	1-3	2.01, -2.35	>.999
Resection weight (g)	843.6 ± 64.12	348-1870	713, 974.2	836.6 [67.60]	252-1788	700.9, 976.3	.761

FMTB, force modulating tissue bridge; SEM, standard error of the mean; SOC, standard of care.

**Table 4.** Study Follow-up During 8-week Intervention Period

Follow-up appointment target visit window	Number of completed visits	Mean [SD] (days)	Range (days)	95% CI (days)	Number of visits (%) within target visit window
Week 2 14 ± 3 days	34 (100)	13.6 [2.2]	9-21	12.7, 14.5	28 (82)
Week 4 28 ± 3 days	31 (91)	28.5 [2.9]	19-37	27.4, 29.5	29 (94)
Week 6 42 ± 3 days	34 (100)	42.68 [2.9]	36-50	41.6, 43.6	29 (85)
Week 8 56 ± 3 days	33 (97)	58.0 [4.7]	53-78	56.3, 59.7	25 (76)

SD, standard deviation.

### Clinical Assessment of T-junction Healing

Of the 68 breasts in the study, 12 (17.6%) had clinically significant T-junction wound dehiscence ≥1 cm^2^ during the 8-week intervention period (Table 5). Of the 12 T-junction wounds, there was an increased rate of T-junction wound dehiscence in SOC breasts (11 breasts, 91.67%) compared with FMTB breasts (1 breast, 8.3%) (Supplemental Figure 1); this difference was statistically significant (*P* = .006). Matched-pairs testing confirmed there was an association between closure used and the risk of wound dehiscence (*P* = .009), with an odds ratio of 0.091 (95% CI, 0.002, 0.625) and a number needed to treat of 3 breasts (ie, approximately 2 patients) (Table 6). Of the 12 breasts with early T-junction wound dehiscence, there was no statistically significant difference between the mean resection weight of the affected breast (836.7 [112.3] g; 95% CI, 589.4, 1084 g) and the contralateral unaffected breast (842.3 [114.7] g; 95% CI, 590.0, 1095 g) (*P* = .94).

**Table 5.** Contingency Table Illustrating Breasts With Clinically Significant T-junction Dehiscence According to the Type of Dermal Closure Used

Type of closure	T-junction wound >1 cm^2^		Total (breasts)
	Yes	No	
Force modulating tissue bridge (experimental)	1	33	34
Subcuticular suture (control)	11	23	34
Total (breasts)	12	55	68

**Table 6.** Matched-Pairs Tabulation of Vertical Incision Wound Outcomes After Experimental (FMTB) vs Control (Subcuticular) Intervention

Wound >1 cm^2^	FMTB			Odds ratio
SOC	Yes	No	Total	0.091 (95% CI, 0.002, 0.625), *P* = .0094
Yes	0	11	11	
No	1	22	23	
Total	1	33	34	

FMTB, force modulating tissue bridge; SOC, standard of care. The *P*-value was calculated with McNemar's test with the continuity correction. Chi-squared equals 6.750 with 1 degree of freedom.

### Nascent Scar Area

**Figure 10.**
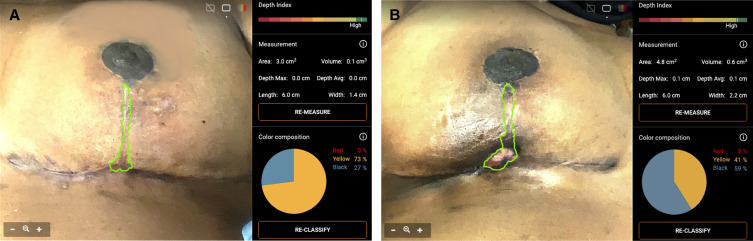
eKare analysis of a vertical incision in a 55-year-old female. (A) Interface for three-dimensional measurement of a vertical incision closed with an FMTB. Metrics included in our analysis included nascent scar area, volume, and maximum depth during the 8-week postoperative intervention period. (B) Interface for three-dimensional imaging analysis of a vertical incision closed according to SOC. Metrics for FMTB and SOC breasts were recorded biweekly and analyzed by paired parametric testing. FMTB, force modulating tissue bridge; SOC, standard of care.

eKare measurements were successfully captured across all visits at Weeks 2, 4, 6, and 8 for 18 patients (Figure 10A, B). The mean nascent scar area was 38.2% lower in the FMTB breast (mean, 1.53 cm^2^) compared with the SOC breast (mean, 2.16 cm^2^) during the 8-week intervention period; this difference was statistically significant (*P* = .0063). When early scar area was stratified according to each postoperative visit, the mean area was lower in the FMTB breast at 2-week (difference, −0.61 cm^2^), 4-week (difference, −0.62 cm^2^), 6-week (difference, −0.35 cm^2^), and 8-week (difference, 0.74 cm^2^) follow-ups compared with the SOC breast (Table 7; Supplemental Figure 2A, B). This difference was statistically significant at 2, 4, and 8 weeks (*P* < .01). The difference was not significantly different at 6 weeks (*P* = .074).

**Table 7.** Nascent Scar Area During the 8-week Postoperative Wound Support and Intervention Period

Time period	Intervention	Mean [SEM] nascent scar area (cm^2^)	95% CI	*P*-value
Overall	FMTB	1.53 [0.10]	1.22, 1.83	.0063
	SOC	2.16 [0.11]	1.80, 2.51	
Week 2	FMTB	1.61 [0.14]	1.30, 1.91	.0053
	SOC	2.28 [0.20]	1.84, 2.72	
Week 4	FMTB	1.69 [0.13]	1.40, 1.97	.0050
	SOC	2.65 [0.17]	2.07, 3.23	
Week 6	FMTB	1.57 [0.10]	1.35, 1.79	.074
	SOC	1.93 [0.25]	1.38, 2.47	
Week 8	FMTB	1.25 [0.17]	0.68, 1.64	.0038
	SOC	2.63 [0.20]	1.95, 3.31	

FMTB, force modulating tissue bridge; SEM, standard error of the mean; SOC, standard of care.

As expected, post hoc analysis showed a statistically significant difference between Week 8 vertical incision nascent scar area in the 11 SOC breasts with clinically significant T-junction dehiscence on exam (Supplemental Figure 3A). Moreover, the area of the affected SOC breast area exceeded that of the contralateral FMTB breast by 0.69 cm^2^ (95% CI, 0.1571, 1.22 cm^2^; *P* = .0087) (Supplemental Figure 3B).

### Multivariate Linear Regression

To determine whether nascent scar area was additionally affected by patient-level variables, we constructed a linear regression model to evaluate whether variables including closure, resection weight, sternal notch-to-nipple distance, Grade 2 or 3 ptosis, race, anesthesia time, or BMI were predictive of variations in nascent scar area at 8-week follow-up. With univariate analysis, Black-American race (*P* = .012), left breast (*P* = .022), and age 18-39 years (*P* = .026) were each independently associated with variations in Week 8 area between closure groups. After adjusting for these variables and others identified in bootstrapping in a multivariate linear regression, only method of closure was significantly associated with variation in Week 8 outcomes (*P* = .0059) (Table 8).

**Table 8.** Multivariate Linear Regression Results, Assessing Relationship Between Predictor Variables and Area at the End of the 2-Month Intervention Period

Patient variable	Week 8 *P*-value
Intercept	.0528
Closure	.0059
Resection weight (g)	.628
Sternal notch to nipple (cm)	.699
Ptosis—Grade 2	.124
Ptosis—Grade 3	.075
Race	.896
Anesthesia time	.435
BMI	.556

Only closure-type was significantly associated with variations in nascent scar area by the end of the intervention period (*P* = .0059).

### Nascent Scar Volume

Overall, mean vertical incision volume (Table 9) was decreased in the FMTB breast (0.1 cm^3^) compared with the SOC breast (0.2 cm^3^) during the 8-week intervention period, and this difference was statistically significant (*P* = .007) (Supplemental Figure 4).

**Table 9.** Mean Vertical Incision Volume During the Postoperative Intervention Period

Time period	Intervention	Mean [SEM] nascent scar volume (cm^3^)	95% CI	*P*-value
Overall	FMTB	0.11 [0.01]	0.04, 0.14	.007
	SOC	0.15 [0.00]	0.14, 0.16	
Week 2	FMTB	0.13 [0.01]	0.11, 0.16	.26
	SOC	0.16 [0.02]	0.12, 0.20	
Week 4	FMTB	0.09 [0.03]	0.03, 0.15	.52
	SOC	0.15 [0.03]	0.09, 0.21	
Week 6	FMTB	0.1 [0.02]	0.06, 0.14	.25
	SOC	0.15 [0.02]	0.10, 0.19	
Week 8	FMTB	0.1 [0.03]	0.04, 0.16	.08
	SOC	0.15 [0.03]	0.08, 0.21	

FMTB, force modulating tissue bridge; SEM, standard error of the mean; SOC, standard of care.

### Nascent Scar Maximum Depth

Similarly, the mean maximum depth (Table 10) of the healing incision was slightly increased in the SOC breast (0.09 cm) compared with the FMTB breast (0.07 cm) but this difference was not statistically significant (*P* = .08) overall.

**Table 10.** Mean Maximum Depth of Nascent Scar Depth During the Postoperative Intervention Period for FMTN Treatment and SOC Closure Breasts

Time period	Intervention	Mean [SEM] nascent scar depth (cm)	95% CI	*P*-value
Overall	FMTB	0.07 [0.01]	0.032, 0.11	.08
	SOC	0.09 [0.00]	0.08, 0.11	
Week 2	FMTB	0.09 [0.01]	0.068, 0.119	.99
	SOC	0.10 [0.00]	0.10, 0.065	
Week 4	FMTB	0.07 [0.02]	0.031, 0.107	.59
	SOC	0.09 [0.01]	0.073, 0.127	
Week 6	FMTB	0.08 [0.01]	0.050, 0.103	.59
	SOC	0.1 [0.01]	0.073, 0.127	
Week 8	FMTB	0.04 [0.02]	−0.006, 0.081	.32
	SOC	0.09 [0.02]	0.034, 0.14	

FMTB, force modulating tissue bridge; SEM, standard error of the mean; SOC, standard of care.

## DISCUSSION

This study provides significant clinical evidence that postoperative mechanomodulation to effectively reduces the incidence of wound complications and the size of healing incisions postoperatively. We hypothesize that, in addition to the benefits of offloading mechanical forces externally, the FMTB helps to maintain adequate perfusion to the distalmost aspects of the lateral and medial flaps, reducing rates of T-junction wound dehiscence during the postoperative period. This difference was further characterized by 3D imaging analysis of the treatment site, which established statistically significant reductions in nascent scar area, depth, and volume in vertical incisions closed with the FMTB.

Although mechanical stress can induce the formation of pathologic scars, it is also important for normal wound healing.^22^ Studies have shown that the tensile strength of tissue after cutaneous injury is linked to the mechanical strength it endures during the wound healing process.^22^ Cremers et al found that nonsplinted excisional wounds closed more quickly than splinted wounds.^23^ They concluded that the removal of mechanical stress delays wound closure 3.3 times in comparison to wounds exposed to mechanical stress.^23^ Other studies have shown that mechanical forces are important for postoperative collagen deposition, neoangiogenesis, and fibroblast migration.^24^ The importance of mechanical stress and loading is the molecular basis of, for example, early mobilization after joint surgery.^11^ Thus, an ideal mechanomodulatory device to support postoperative wound healing might attenuate incisional tension to reduce the risk of complications without completely removing mechanical stress.

Mechanomodulation additionally plays a role in wound healing; innovation and novel techniques to improve postoperative wound outcomes are also warranted, especially in aesthetic surgery where wound morbidity can predict scar cosmesis. One device, the Zipline (Zipline Medical, Inc., Styrker Corporation, Kalamazoo, MI), approximates skin edges after surgery and offsets skin tension.^25^ Like the FMTB, it can also be used as an alternative to superficial sutures and is positioned immediately after surgery. Clinical studies either did not establish differences or superiority in wound outcomes or concluded the device was contraindicated in high-tension wounds, but established improvements in scar outcomes.^26-28^ A 2020 meta-analysis comparing the Zipline to suture closure found a decreased rate of skin/soft tissue infection and decreased incision closure time but did not find a significant difference in rates of dehiscence.^25^ Additionally, the study did not involve patients undergoing breast surgery.

Other hypothesized contributors to incisional dehiscence besides excess mechanical stress include variations in surgical technique and low relative perfusion at the healing site.^29^ Because patients in this study were self-controlled, we considered the role of the surgeon’s technique to be negligible. Additionally, the unmasking of randomization only occurred after approximation and closure of the deep tissue layers, when final dermal closure was to begin. The role of perfusion, however, has been described as a contributor to early wound dehiscence in elective cosmetic and body contouring procedures.^29^ For example, a recent study showed rates of clinically meaningful wound dehiscence were higher in a breasts undergoing standard dressing after Wise-pattern reduction mammaplasty compared with those receiving closed incisional negative-pressure therapy for 6 days after surgery.^29^ Similar to our study, of 15 incidences of wound dehiscence, the authors found 14 (93%) occurred in breasts undergoing SOC dressing, while 1 (7%) occurred in a breast treated postoperatively with closed incisional negative-pressure therapy; however, this study was not self-controlled.^29^ As studies have shown that negative-pressure wound therapy supports cutaneous microcirculation and perfusion after injury, the reduced rate of dehiscence in this study is particularly interesting.^30,31^ However, Cochrane reviews assessing negative-pressure therapy across surgical subspecialties have shown a low level of evidence for postoperative wound healing.^32,33^ Distal flap edges are at risk for epidermolysis or necrosis in breast surgery due to their relative distance from the pedicle or random-pattern blood supply,^34-36^ and the resulting poor tissue quality can cause dehiscence.^37^ Sutures also have the potential to contribute to relative tissue ischemia.^38^

Given the myriad variables which influence wound healing and scar formation—ie, genetics, environment, nutrition, ethnicity, anatomic location—within-subject controls allowed objective comparison of wound healing and scar quality between breasts in the context of the individual patient. The reduction mammaplasty was chosen as the clinical model due to the frequency of the procedure, the relative ease of experimental device application, the ability for bilateral assessment of experimental closure and control, and because the vertical incision was a surgical site of relatively uncomplicated healing.

The area, volume, and depth of healing incisions closed by FMTBs were significantly decreased overall compared with SOC closure during the postoperative intervention period. The omnipresence of early T-junction dehiscence during the postoperative period presents a vexing challenge for plastic surgeons.^39^ Overall, our decreased rate of wound dehiscence in the FMTB breast (1.47%) marks a significant statistical and clinical departure from rates reported in the literature.^40^ Additionally, after adjusting for other patient variables, we found only closure significantly affected variations in the area of healing incisions. Outcome studies of Wise-pattern resections have reported rates of minor wound dehiscence of between 8% and 100%, citing patient variables such as BMI and resection weight as contributors.^40-45^ A recent retrospective study identified a relationship between BMI and wound healing greater than 2 months.^46^ The reduced rates of dehiscence and smaller nascent scar areas observed with this device after controlling for key variables, including race, resection weight, BMI, and ptosis, suggest its clinical efficacy. Additionally, with a calculated number needed to treat of 3 breasts, 2 patients treated with incisions closed with FMTBs would prevent the occurrence of 1 dehisced incision, indicating the potential to significantly support clinical outcomes in this patient population.

Our study is not without limitations. First, as visits took place during the COVID-19 pandemic, patients were only able to attend Week 4 and Week 6 visits virtually, where they were clinically examined remotely and were supervised in the removal and replacement of FMTBs. This upheld patient autonomy and comfort. At these time points, if patients attended virtually, 3D measurement data were not collected. Thus, we report findings from those patients able to attend all 4 visits during the 8-week intervention period. Second, a brief learning curve is required to use the FMTB effectively. Patients worked with investigators to gain familiarity with the device during initial screening visits in a hands-on practice session. Each subject received multiple packs prior to surgery with which they could practice or use to teach loved ones potentially helping with postoperative care. Additionally, the nascent scar depth data calculated from the eKare data have lower inter- and intrarater reliability compared with nascent scar area and volume measurements, according to published reports. The data presented here evaluate postoperative wound healing and early scar measures and represent completed analysis from the first 8 weeks of this study. The study is powered to enroll up to 42 participants for an assessment of POSAS whereby patients are continually followed through 12 months to assess long-term scar cosmesis and scar outcomes. The entirety of the study is anticipated to continue until spring 2024, and findings regarding scar quality and cosmesis will be reported following study completion. Other studies may build upon ours by comparing the clinical efficacy of FMTBs directly to other mechanomodulatory devices and adjuncts, expanding this study model to multiple centers.

## CONCLUSIONS

FMTBs are a novel adjunct for postoperative mechanomodulation to mitigate wound healing complications. Preliminary findings from a within-patient, randomized controlled trial showed that FMTBs significantly decrease nascent scar area and the rate of T-junction dehiscence after Wise-pattern reduction mammaplasty. These findings suggest the FMTB, by reducing transverse tissue strain, potentially relieves the cutaneous and subcutaneous tension and distal flap relative ischemia that typically contribute to T-junction dehiscence in this clinical model. Further research should build on this study by analyzing the efficacy of this device in a larger study population and in patients undergoing other reconstructive procedures.

## Supplemental Material

This article contains supplemental material located online at www.aestheticsurgeryjournal.com.

## Supplementary Material

sjad269_Supplementary_Data
