# Supplementary material for: Postoperative Mechanomodulation Decreases T-Junction Dehiscence After Reduction Mammaplasty: Early Scar Analysis From a Randomized Controlled Trial
Source: Aesthet Surg J. 2023 Aug 22;43(12):NP1033–48. doi: 10.1093/asj/sjad269 (PMC10902896; doi:10.1093/asj/sjad269)
Supplement: sjad269_Supplementary_Data [file sjad269_Supplementary_Data.zip › 23-0639_Figure Legends.docx]

**Supplemental Figure Legends**

**Supplemental Figure 1.** (A) Clinical assessment showed a statistically significant, increased rate of early T-junction wound dehiscence in the SOC breast compared to the FMTB breast after paired binomial analysis (p=.0063). **p<.01. (B) A 27-year-old female patient at 4-week follow-up. Clinical measurement of T-junction wound dehiscence in breast closed using FMTB. Only one breast was affected by wound dehiscence > 1 cm2 after closure by FMTB vs 11 breasts undergoing SOC closure. (p=.0063). FMTB, force modulating tissue bridge; SOC, standard of care.

**Supplemental Figure 2.** (A) Mean nascent scar area during 8-week postoperative intervention period when force modulating tissue bridges were continuously applied. The area of the healing nascent scar was significantly decreased in the breast treated with force modulating tissue bridges (p=.0063). (B) Nascent scar area during intervention period stratified according to postoperative week. Mean area was significantly lower at 2- (p=.005), 4- (p=.0005), and 8-week follow-up (p=.004) in the vertical incision closed using force-modulating tissue bridges compared to the standard of care closure breast. **p< .01. FMTB, force modulating tissue bridge; SOC, standard of care.

**Supplemental Figure 3.** (A) 3D analysis of healing incision area in patients with clinically significant T-junction dehiscence. Consistent with other findings, post-hoc analysis showed larger mean scar area in those SOC breasts affected by dehiscence compared to the contralateral breast closed using FMTB. (p=.0087). (B) Estimation plot showing paired differences between nascent scar area in SOC breasts with T-junction dehiscence versus the contralateral, unaffected breast closed using SOC technique. FMTB, force modulating tissue bridge; SOC, standard of care.

**Supplemental Figure 4.** Mean nascent scar volume over study period. Mean nascent scar volume was significantly reduced (p=.007) after closure with force modulating tissue bridges compared to those closed using standard of care subcuticular technique. FMTB, force modulating tissue bridge; SOC, standard of care.
