# Supplementary figures and images for: Postoperative Mechanomodulation Decreases T-Junction Dehiscence After Reduction Mammaplasty: Early Scar Analysis From a Randomized Controlled Trial
Source: Aesthet Surg J. 2023 Aug 22;43(12):NP1033–48. doi: 10.1093/asj/sjad269 (PMC10902896; doi:10.1093/asj/sjad269)

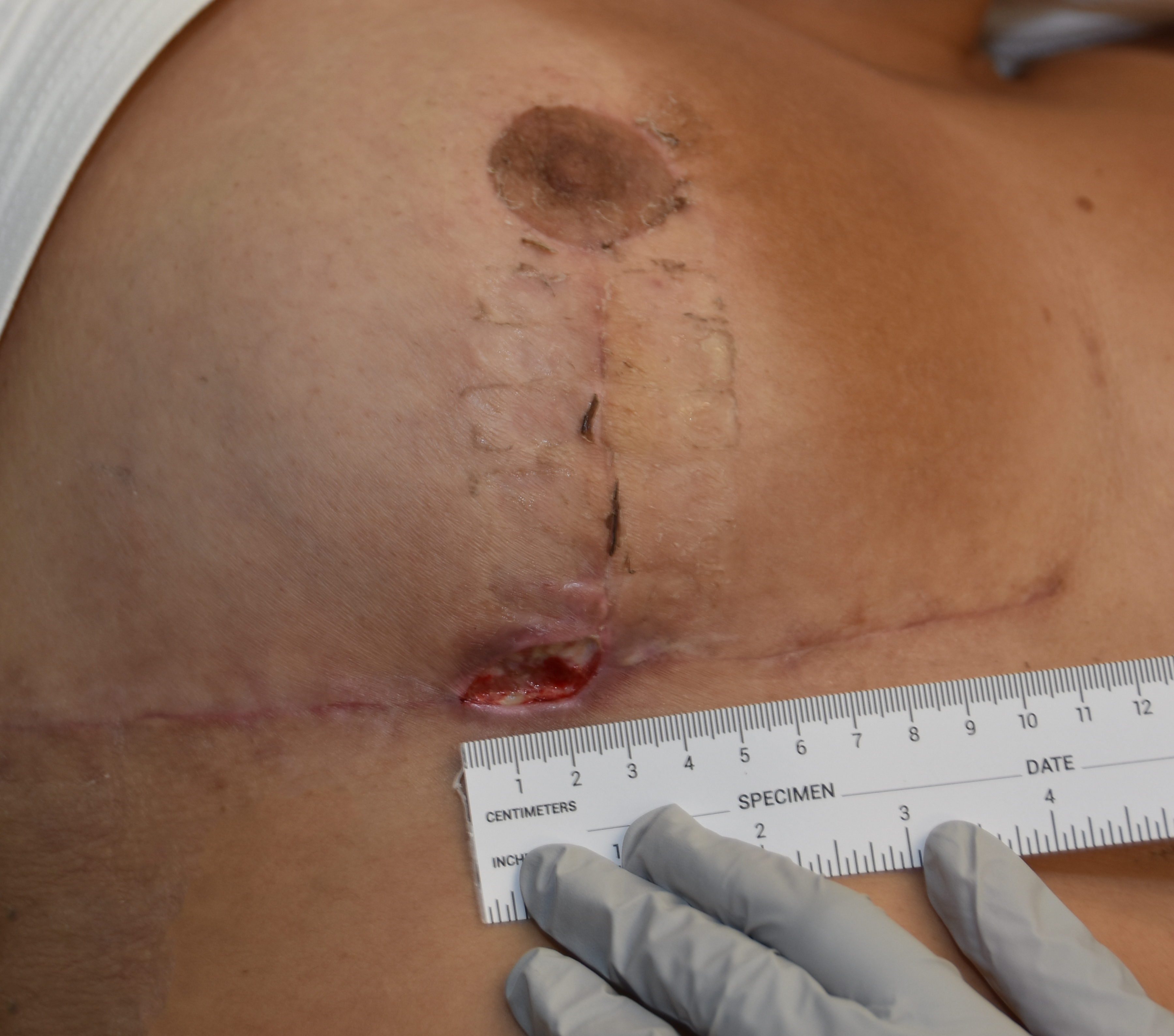

Supplement: sjad269_Supplementary_Data [file sjad269_Supplementary_Data.zip › Supplemental Figure 1b_Clinical measurement.jpg]

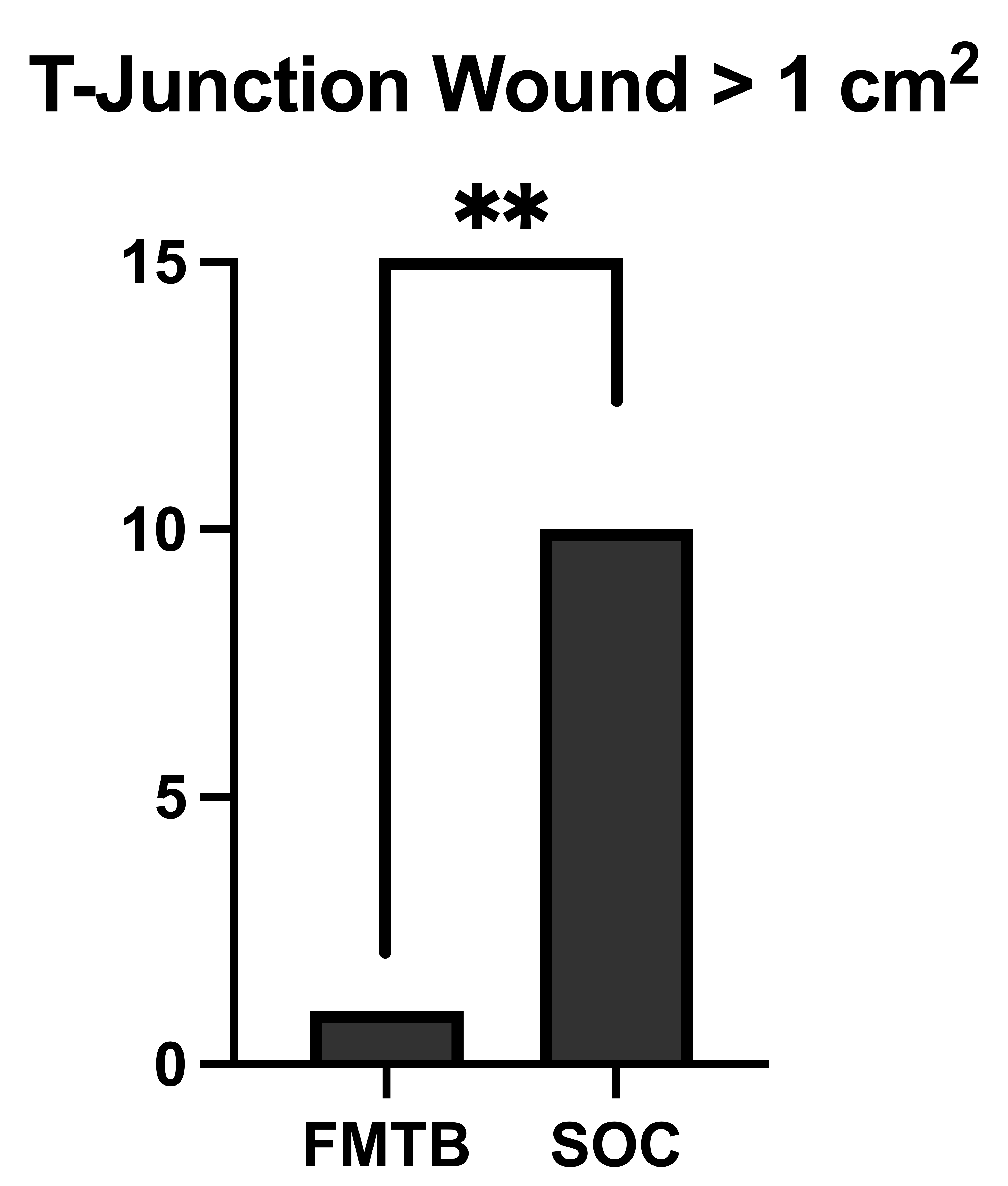

Supplement: sjad269_Supplementary_Data [file sjad269_Supplementary_Data.zip › Supplemental Figure 1_Binomial Testing for Wound _ 1cm [Wound _ 1cm].png]

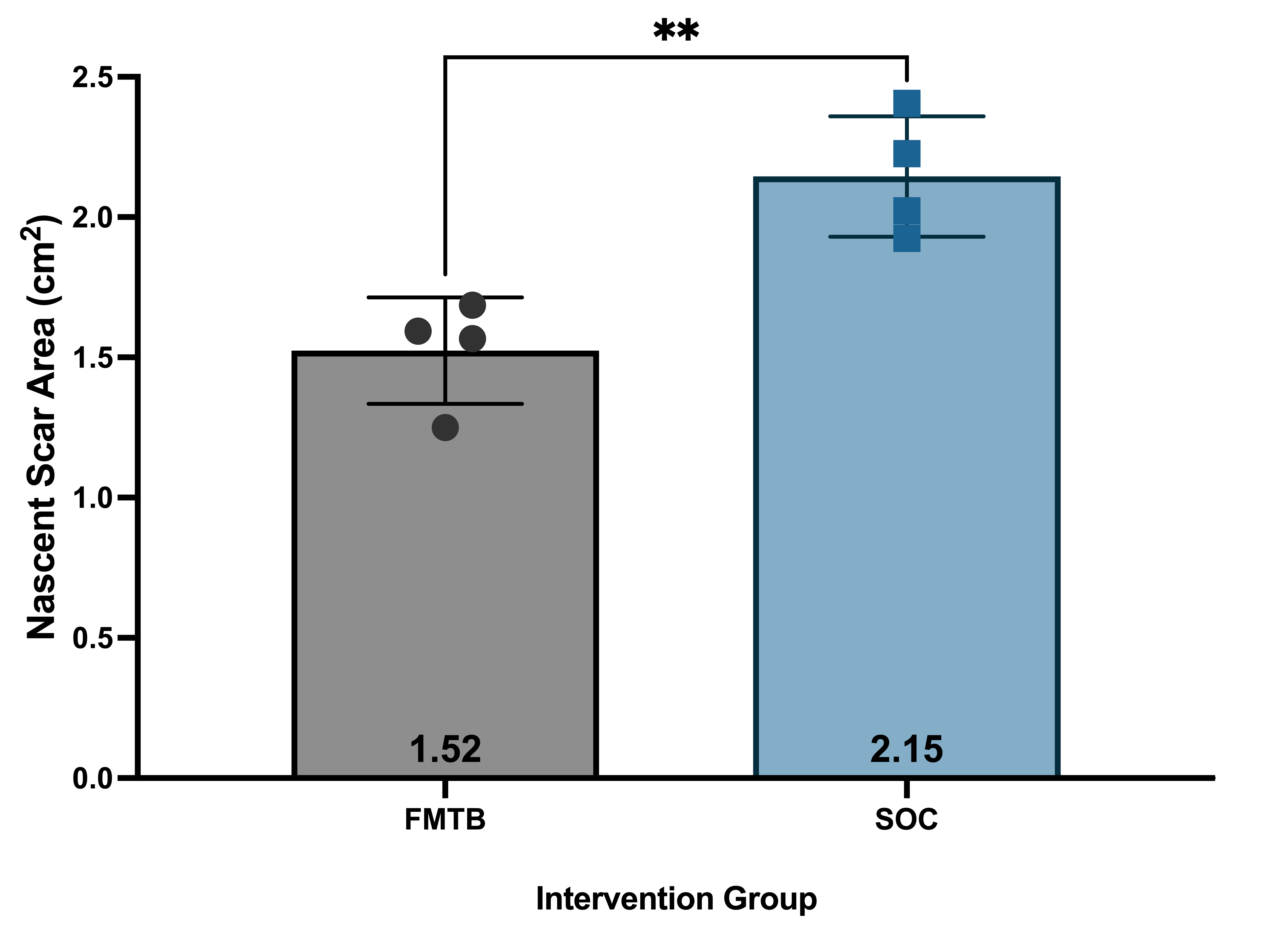

Supplement: sjad269_Supplementary_Data [file sjad269_Supplementary_Data.zip › Supplemental Figure 2a_eKare Measurements - Area.png]

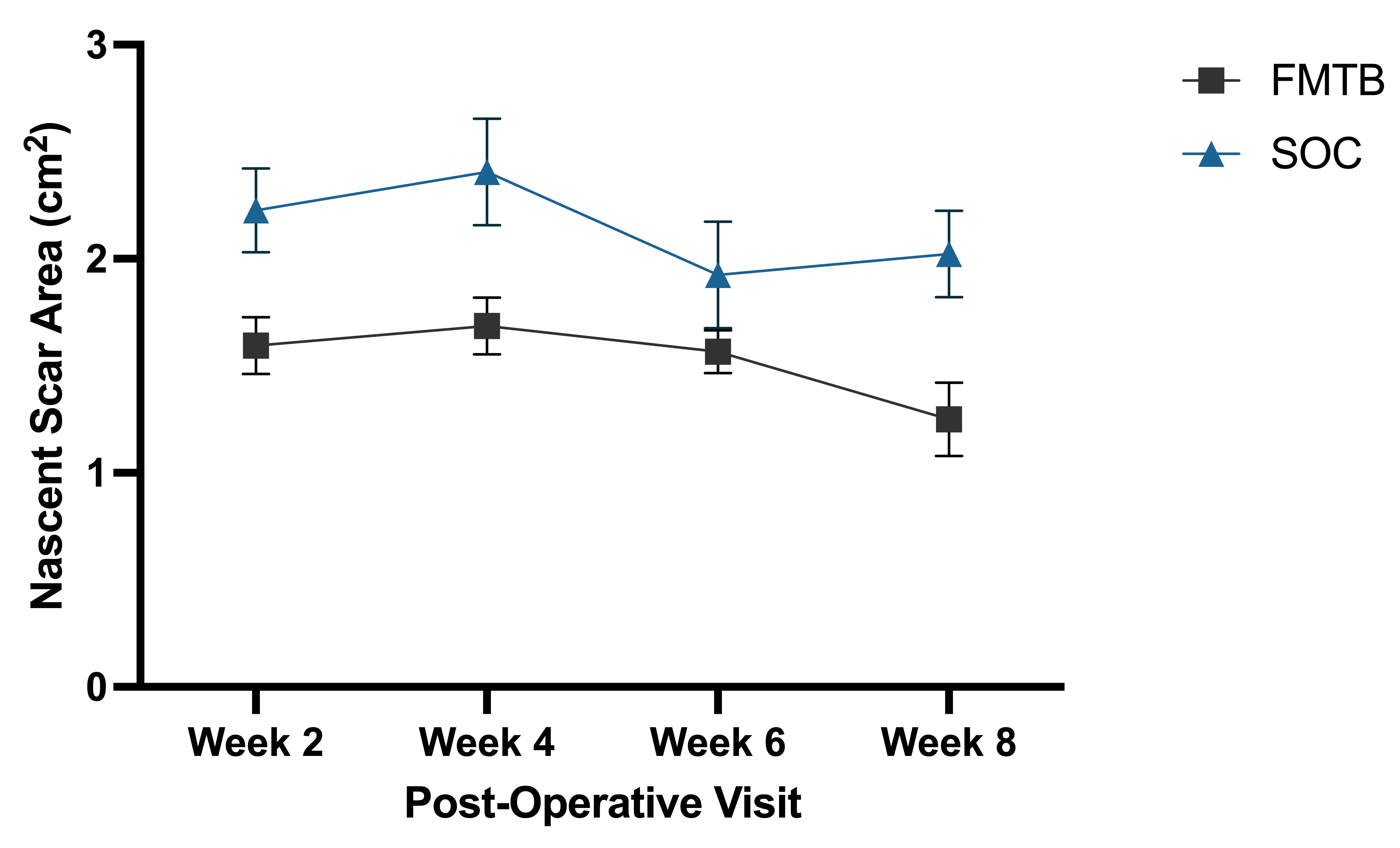

Supplement: sjad269_Supplementary_Data [file sjad269_Supplementary_Data.zip › Supplemental Figure 2b_eKare Measurements - Area Over Time.png]

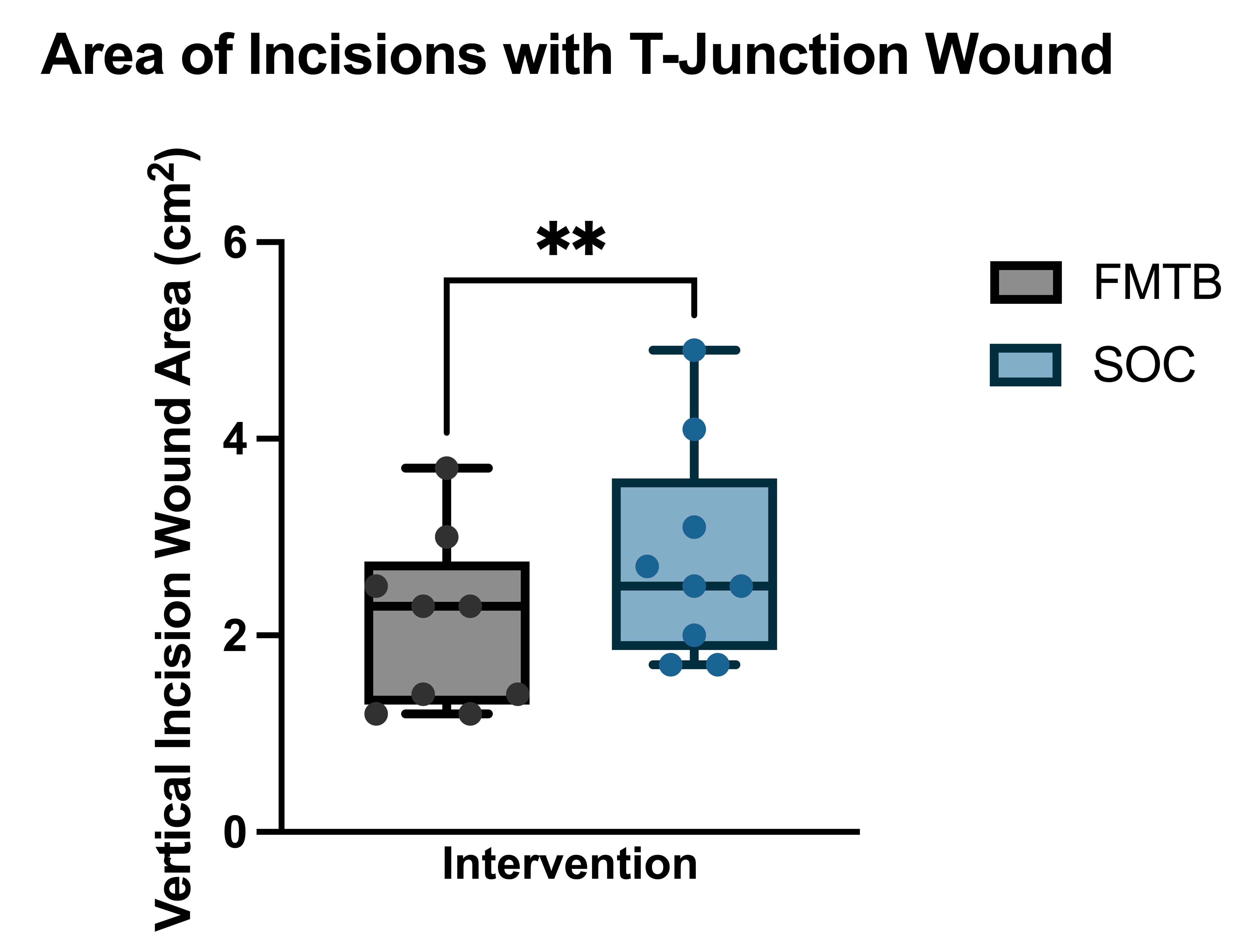

Supplement: sjad269_Supplementary_Data [file sjad269_Supplementary_Data.zip › Supplemental Figure 3a_Vertical Incision Area in SOC breasts with wounds.png]

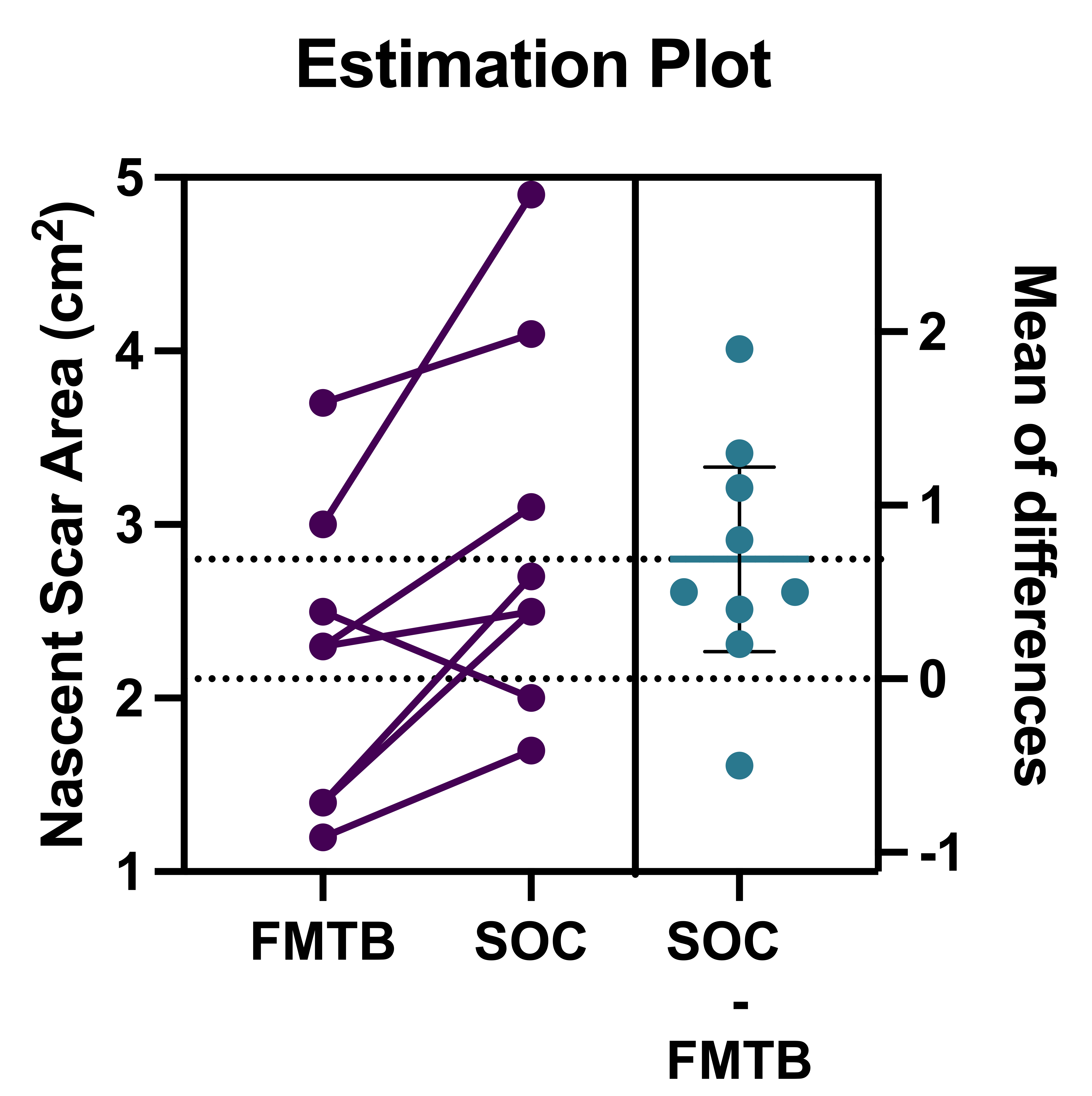

Supplement: sjad269_Supplementary_Data [file sjad269_Supplementary_Data.zip › Supplemental Figure 3b_Estimation Plot- Paired t test of Vertical Incision Area in SOC breasts with wounds.png]

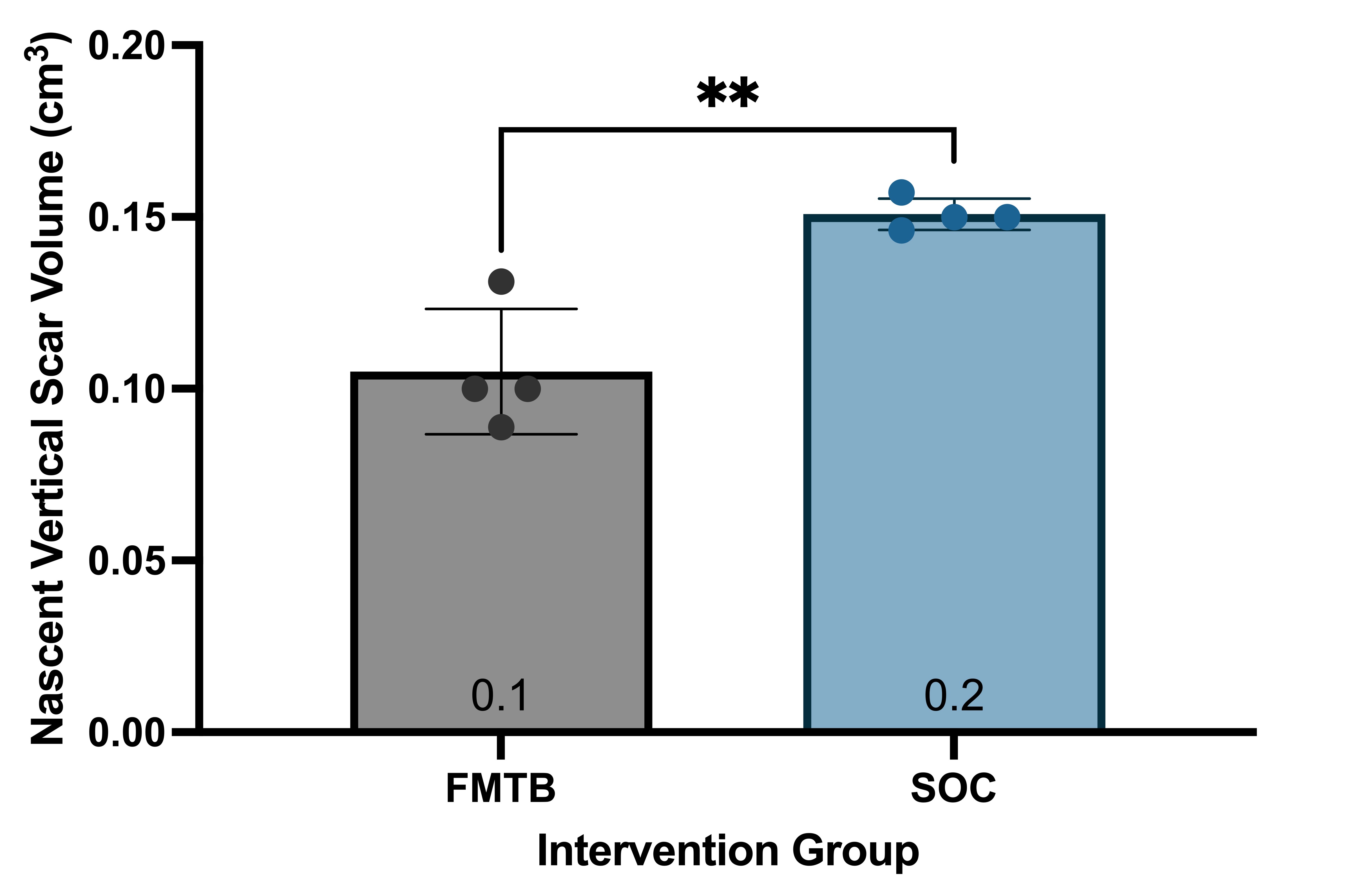

Supplement: sjad269_Supplementary_Data [file sjad269_Supplementary_Data.zip › Supplemental Figure 4_eKare - Volume Overall.png]
